# Morphology-based deep learning enables accurate detection of senescence in mesenchymal stem cell cultures

**DOI:** 10.1186/s12915-023-01780-2

**Published:** 2024-01-02

**Authors:** Liangge He, Mingzhu Li, Xinglie Wang, Xiaoyan Wu, Guanghui Yue, Tianfu Wang, Yan Zhou, Baiying Lei, Guangqian Zhou

**Affiliations:** 1 Guangdong Key Laboratory for Biomedical Measurements and Ultrasound Imaging, National-Regional Key Technology Engineering Laboratory for Medical Ultrasound, School of Biomedical Engineering, Shenzhen University Medical School, 1066 Xueyuan Avenue, Shenzhen, 518060 China; 2 Department of Medical Cell Biology and Genetics, Shenzhen Key Laboratory of Anti-Aging and Regenerative Medicine, Shenzhen Engineering Laboratory of Regenerative Technologies for Orthopedic Diseases, Shenzhen University Medical School, Shenzhen, 518060 China; 3 Department of Dermatology, Shenzhen Institute of Translational Medicine, Shenzhen Second People’s Hospital, The First Affiliated Hospital of Shenzhen University, Shenzhen, 518035 China; 4 Lungene Biotech Ltd., Shenzhen, 18000 China

**Keywords:** Mesenchymal stem cells, Senescence, Deep learning, Morphology

## Abstract

**Background:**

Cell senescence is a sign of aging and plays a significant role in the pathogenesis of age-related disorders. For cell therapy, senescence may compromise the quality and efficacy of cells, posing potential safety risks. Mesenchymal stem cells (MSCs) are currently undergoing extensive research for cell therapy, thus necessitating the development of effective methods to evaluate senescence. Senescent MSCs exhibit distinctive morphology that can be used for detection. However, morphological assessment during MSC production is often subjective and uncertain. New tools are required for the reliable evaluation of senescent single cells on a large scale in live imaging of MSCs.

**Results:**

We have developed a successful morphology-based Cascade region-based convolution neural network (Cascade R-CNN) system for detecting senescent MSCs, which can automatically locate single cells of different sizes and shapes in multicellular images and assess their senescence state. Additionally, we tested the applicability of the Cascade R-CNN system for MSC senescence and examined the correlation between morphological changes with other senescence indicators.

**Conclusions:**

This deep learning has been applied for the first time to detect senescent MSCs, showing promising performance in both chronic and acute MSC senescence. The system can be a labor-saving and cost-effective option for screening MSC culture conditions and anti-aging drugs, as well as providing a powerful tool for non-invasive and real-time morphological image analysis integrated into cell production.

**Supplementary Information:**

The online version contains supplementary material available at 10.1186/s12915-023-01780-2.

## Background

Mesenchymal stem cells (MSCs) are one of the fundamental therapeutic tools in the fields of regenerative medicine and tissue engineering [1]. MSCs are considered an ideal candidate for replacing lost or damaged cells and tissues in vivo, as they possess the capacity for multi-lineage differentiation and self-renewal, along with secreting various factors that exhibit pro-angiogenic, immunomodulatory, and anti-apoptotic functions. Their therapeutic efficacy has been demonstrated in numerous clinical trials [2, 3].

As adult stem cells, MSCs exhibit a shorter lifespan compared to pluripotent stem cells [4]. Cell senescence, which represents the fundamental process of aging, occurs throughout the life cycle. In vivo, cell senescence serves as a mechanism for the organism to inhibit the proliferation of damaged cells and thus prevent their progression toward oncogenic transformation [5]. However, senescent MSCs exhibit persistent growth arrest, decreased self-renewal capacity, and an inflammatory phenotype, which can lead to tissue regeneration damage and organ degeneration [6]. In vitro, cell senescence can compromise the differentiation and immunosuppressive properties of MSCs, impair their normal function, increase their susceptibility to genetic instability, and alter the microenvironment through senescence-associated secretory phenotype (SASP) [7]. The senescent MSCs exhibit an augmented cell size and a shift in morphology from spindle to an irregular flat shape, attributed to the accumulation of undegraded macromolecules. In addition, senescent cells exhibit cell division fatigue, accumulation of senescence-associated β-galactosidase (SA-β-gal), nuclear heterochromatin accumulation, and increased expression of cyclin-dependent kinase inhibitors (p16 and p21) [8].

In the fields of tissue engineering and cell therapy, the large-scale and long-term culture required for MSCs can result in replicative senescence and impaired cell quality and function through continuous expansion in vitro, which poses potential safety risks [9]. Therefore, effective assessment of senescence is crucial prior to the application of cell therapy. The ideal method for detecting MSC senescence should be quantitative, rapid, label-free, and non-destructive methodologies. Currently, a range of biological and spectroscopic techniques can be employed to assess cell senescence based on indicators such as SA-β-gal [10, 11], telomere length [12], epigenetic markers [13], gene and protein markers [14], as well as cell fluorescence intensity [15, 16]. However, these methods pose challenges for real-time monitoring or may cause irreversible modification or even destruction of cells during detection.

Senescent cells display distinct morphology, which can serve as a means to assess their senescence status. Developing morphology-based techniques for evaluating cell quality by exploiting the differences in appearance between senescent and non-senescent MSCs holds great promise and potential applications. Experienced researchers are capable of evaluating the cell state based on its morphological characteristics. However, uncertainties may arise in mass microscopic observations due to researcher factors such as fatigue, efficiency, experience level, and subjective elements. The emergence of deep learning technology provides a new approach for analyzing cellular images [17, 18]. The deep learning-based object detection algorithm possesses robust localization and classification capabilities for objects in a scene, enabling automatic feature extraction of cell images and simultaneous prediction of cell position and category within the entire image, thereby facilitating automated analysis of cellular imagery [19–21]. The region-based convolution neural network (R-CNN) is a classic representative of an object detection network that has been widely applied in the field of medical image processing [22]. Following the development of R-CNN, subsequent advancements in object detection algorithms have included the introduction of Fast R-CNN [23], Faster R-CNN [24], and Cascade R-CNN [25]. The Cascade R-CNN serves as a versatile framework for the R-CNN series detectors, exhibiting the potential to enhance the performance of various R-CNN detectors such as Faster R-CNN, Mask R-CNN, and deformable region-based fully convolutional network (Deformable R-FCN) by 2–4%. Moreover, it exhibits promising capabilities in analyzing pathological images [25–27]. Therefore, Cascade R-CNN holds promise in developing a reliable real-time tool for assessing the MSC status. In this study, we have developed a novel Cascade R-CNN system based on cell morphology for the detection and precise localization of single senescent cells, as well as the evaluation of induced pluripotent stem cell-derived MSCs (iMSCs) in microscopic image. Furthermore, we assessed the suitability of the Cascade R-CNN system for intervening in senescence and examined the correlation between morphological changes and other senescence markers. The combination of Cascade R-CNN based on cell morphology and conventional optical microscopy has the potential to replace traditional biological and spectroscopy techniques in certain applications, offering a cost-effective and labor-saving alternative for screening MSC culture conditions and anti-aging drugs, thereby contributing to the industrialization of MSC.

## Results

### Replicative senescence of iMSCs

**Fig. 1:**
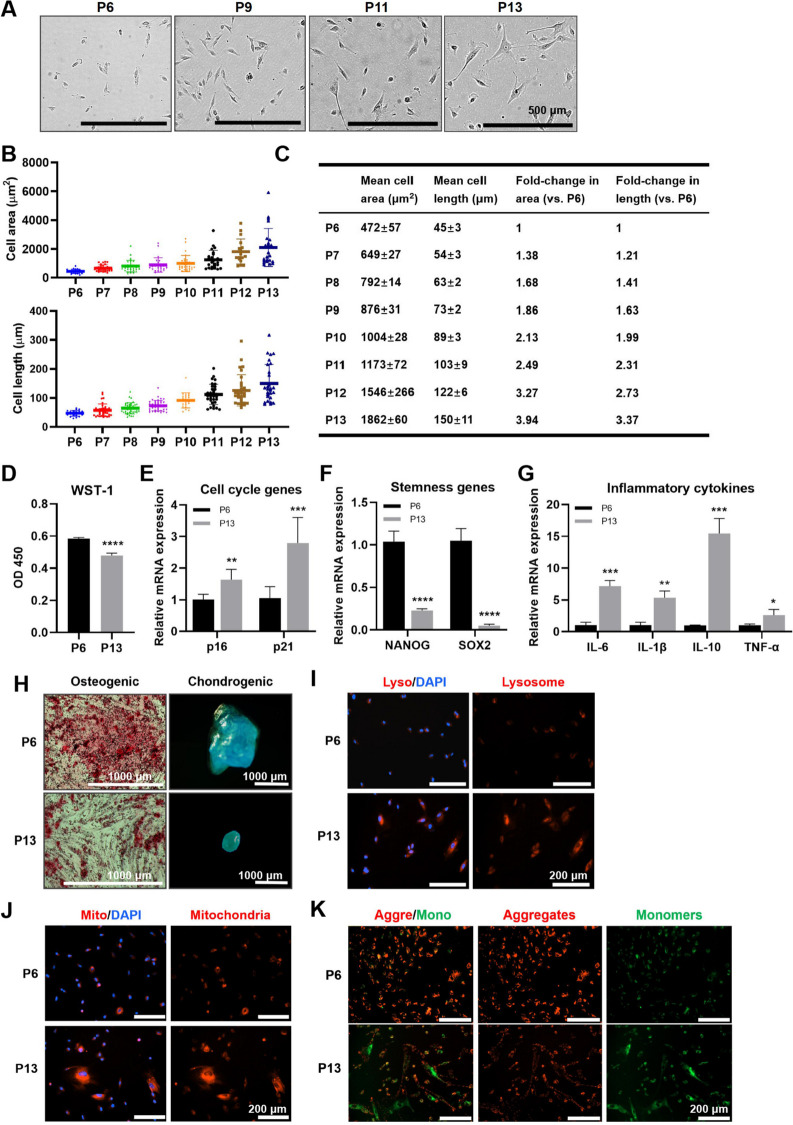
Senescent markers and stemness properties of iMSCs changed with serial culture. **A** Representative morphology of long-term iMSCs from passages 6, 9, 11, and 13 imaged. Scale bar: 500 μm. **B** Mean cell area and length increased with extended in vitro cell culture. **C** Mean cell areas, corresponding cell length, and fold-changes in comparison to P6. **D** Proliferation of iMSCs at P6 and P13. The mRNA levels of cell cycle genes (**E**), stemness genes (**F**), and inflammatory cytokines (**G**) of iMSCs at P6 and P13. **H** Osteogenic and chondrogenic differentiation of iMSCs at P6 and P13. Scale bar: 1000 μm. **I** The lysosomal density of iMSCs at P6 and P13. Scale bar: 200 μm. **J** The mitochondrial density of iMSCs at P6 and P13. Scale bar: 200 μm. **K** The MMP of iMSC at P6 and P13. Scale bar: 200 μm. Data were representative of over three independent experiments. *n* = 3, * *p* < 0.05; ** *p* < 0.01; *** *p* < 0.001; **** *p* < 0.0001 by Student’s *t* test

We cultured human iMSCs in vitro for an extended period, and the cells initially exhibited senescence characteristics at the 6th passage, which became increasingly apparent with continued passaging. The replicative senescence induced by external culture occurs gradually and at a slow pace. We conducted a comparative analysis of cellular morphological characteristics from passage 6 (P6) to passage 13 (P13), revealing an irregular cell morphology (Fig. 1A), accompanied by a gradual increase in both cell area and length (Fig. 1B,C). Subsequently, other senescent-related indices were compared between P6 (considered as early senescence) and P13 (considered as late senescence) cells. It was observed that continuous passaging resulted in decreased cell proliferation (Fig. 1D), upregulated expression of cell cycle genes (*p16* and *p21*; Fig. 1E), downregulated expression of stemness genes (*NANOG* and *SOX2*; Fig. 1F), increased expression of inflammatory cytokine genes (*interleukin 6* [*IL-6*], *IL-1β*, *IL-10*, and *tumor necrosis factor-α* [*TNF-α*]; Fig. 1G), weakened osteogenic and chondrogenic differentiation potential (Fig. 1H), elevated lysosome and mitochondrial density levels (Fig. 1I-J), and altered mitochondrial membrane potential (MMP; Fig. 1K). Except for morphology, these indicators could reflect the senescent trend across different generations; however, the detection process caused irreversible damage to cells and yielded inconsistent results between successive generations. Notably, cell size and morphology were easily observable indicators of cell state, implying that quantification and analysis of senescent-related morphological changes would be advantageous for developing powerful and straightforward methods to detect senescent cells.

### A Cascade R-CNN system detected senescent cells with promising performance

**Fig. 2:**
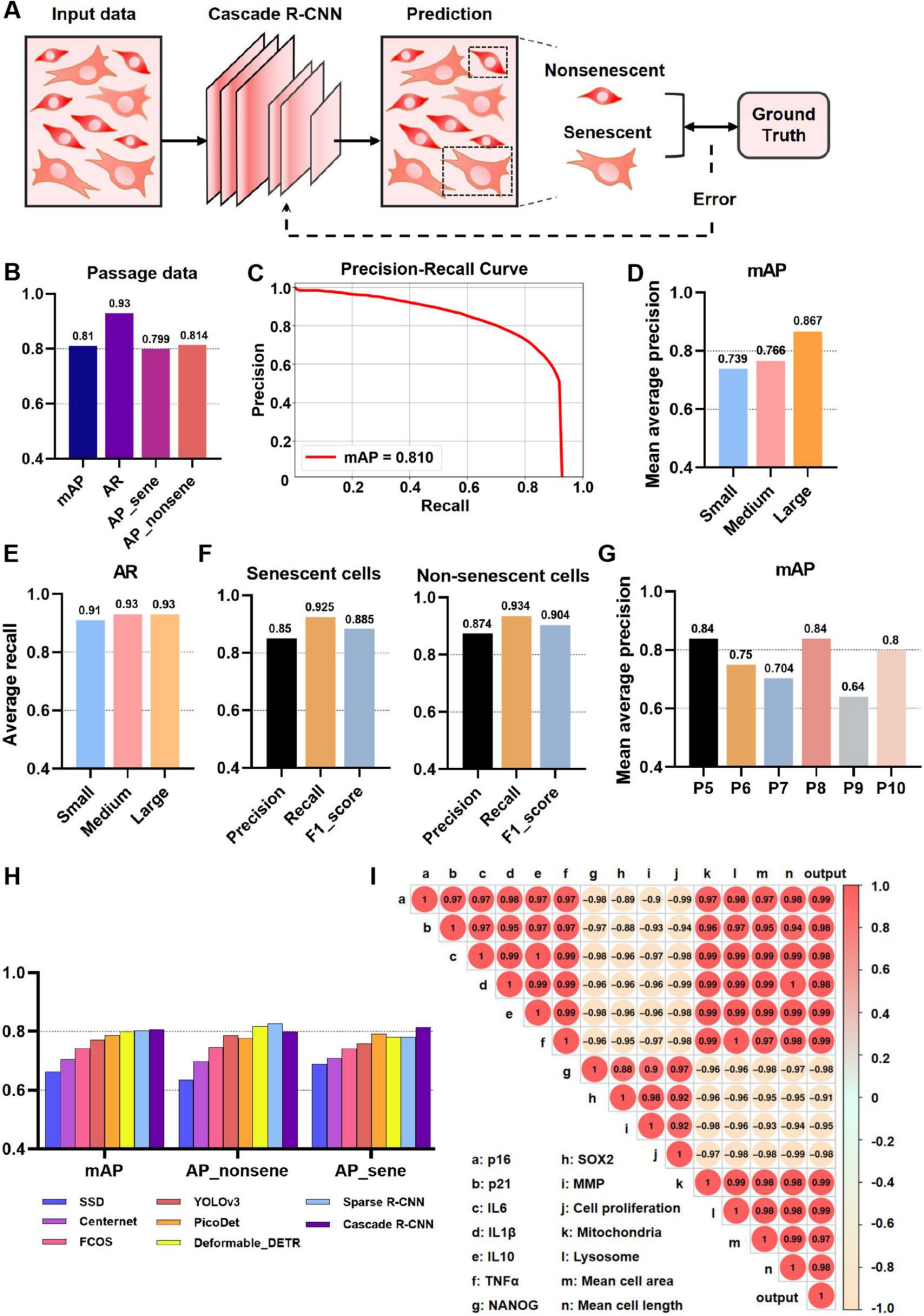
Cascade R-CNN training to distinguish non-senescent and senescent cells. **A** Concept of the Cascade R-CNN system. Microscopic images of iMSCs during culture were acquired, and the Cascade R-CNN was trained to detect non-senescent and senescent cells. **B** The AP, mAP, and AR showed the performance of the Cascade R-CNN trained by passage data. **C** Precision-recall curve of the trained Cascade R-CNN. **D** The mAP of the small, medium, and large objects. **E** The AR of the small, medium, and large objects. **F** Several evaluation indexes for non-senescent and senescent cell detection. **G** The performance of the Cascade R-CNN in serial passages. **H** The performance of deep learning methods. **I** The Pearson correlation coefficient between the senescence proportion output of the Cascade R-CNN and senescence-related indicators. Data were representative of three independent experiments, *n* = 3

Cell morphology serves as a specific indicator for distinguishing cell states. Researchers use morphology to identify the cell state but often produce uncertain detection results when faced with mass microscopic observation tasks. Developing an automated detection method for bright-field microscopic cell images based on senescence-related cell morphology offers significant advantages and promising application prospects. We prepared an input dataset consisting of 640 × 640-pixel bright-field images at a magnification of × 4. The images were performed three times independently for each passage to enhance data generalization. Following pretreatment, experienced researchers classified and labeled the cells in the images based on SA-β-gal staining activity and cell morphology (Fig. S1A). The training dataset consisted of 2382 images, while the validation and test datasets contained 797 and 807 images. The training dataset consisted of 56,115 senescent cells and 27,550 non-senescent cells. The validation dataset contained 18,945 senescent cells and 9284 non-senescent cells. Similarly, the test dataset included 18,847 senescent cells and 9284 non-senescent cells. Subsequently, a Cascade R-CNN network was employed to evaluate whether the cells were senescent or non-senescent (Fig. 2A). To improve precision, predictions were compared with ground truth, and weights were iteratively adjusted automatically to train the Cascade R-CNN.

We assessed the capacity of the network to distinguish between non-senescent and senescent cells throughout successive passages (P5 to P10) in culture. After training, the Cascade R-CNN network demonstrated high precision in detecting both senescent and non-senescent cells. For network evaluation, precision, recall, *F*1 score, PR curve, average precision (AP), mean average precision (mAP), and average recall (AR) were utilized with mAP50 as the test index. Precision represents the positive prediction hit rate while recall indicates prediction sensitivity. The *F*1 score combines both precision and recall. The precision-recall (PR) curve reflects the trade-off between precision and recall with better performance indicated by a large area under the curve. The AP represents the area under the PR curve and serves as a measure of the average precision of the network in detecting a specific object category. The mAP, which is calculated as the mean of all APs across different categories, is considered to be the most crucial metric for evaluating object detection performance. The AR refers to the ratio of correctly detected objects to total detected objects in a given test dataset. The trained Cascade R-CNN network exhibited robust performance, achieving a mAP of 0.81 and an AR of 0.93. The AP for senescent cells and non-senescent cells were 0.799 and 0.814, respectively (Fig. 2B,C). The mAP values of objects of small size (object box area under 32^2^ pixels), medium size (object box area between 32^2^ and 96^2^ pixels), and large size (object box area beyond 96^2^ pixels) were found to be 0.739, 0.766, and 0.867, respectively (Fig. 2D). The AR values of small, medium, and large objects were 0.91, 0.93, and 0.93, respectively (Fig. 2E). Regarding senescent cells, the precision, recall, and *F*1 score achieved were 0.850, 0.923, and 0.885, respectively. As for non-senescent cells, the precision, recall, and *F*1 score were 0.874, 0.934, and 0.903, respectively (Fig. 2F). The capacity of the network to distinguish between non-senescent and senescent cells was subsequently evaluated at each passage. The representative images were randomly selected from each passage (P5 to P10). Notably, the trained Cascade R-CNN network exhibited varying performances across passages, demonstrating robust performance in passages 5, 8, and 10, with mAP values of 0.84, 0.84, and 0.8, respectively (Fig. 2G and Fig. S1B-C). These findings provided compelling evidence for the successful implementation of an object detection system for serial culture-induced senescent iMSCs.

To evaluate the superiority of the Cascade R-CNN network, we compared these findings with feature-based deep learning approaches, including the single shot multibox detector (SSD) [28], CenterNet [29], full convolutional one-stage object detection (FCOS) [30], you only look once v3 (YOLOv3) [31], PicoDet [32], deformable detection transformer (Deformable DETR) [33], and Sparse R-CNN [34]. We employed deep learning models to analyze cell images by extracting features of picture attributes, resulting in input datasets. However, the mAP values obtained from these models were comparatively lower than those achieved by the Cascade R-CNN network. Therefore, we determined that the Cascade R-CNN network was the optimal approach for our study (Fig. 2H).

Distinct morphological features were observed in senescent cells, and the proportion of senescent cells was determined by quantifying both senescent and non-senescent cells. The correlation between the output of the Cascade R-CNN system for senescence proportion and various markers associated with cell senescence was evaluated using the Pearson correlation coefficient to analyze changes in different parameters related to cell senescence. Strong positive correlations were observed between senescence proportion output and cell cycle genes (*p16* and *p21*), inflammatory cytokines (*IL-6*, *IL-1β*, *IL-10*, and *TNF-α*), the density of lysosome and mitochondrial, cell area, and cell length. Conversely, strong negative correlations were found between senescence proportion output and stemness genes (*NANOG* and *SOX2*), MMP, and cell proliferation (Fig. 2I). These findings suggested that the morphology-based method was a reliable approach for assessing MSC senescence comparable to other markers.

### Cascade R-CNN system performance at doxorubicin-induced iMSC senescence

**Fig. 3:**
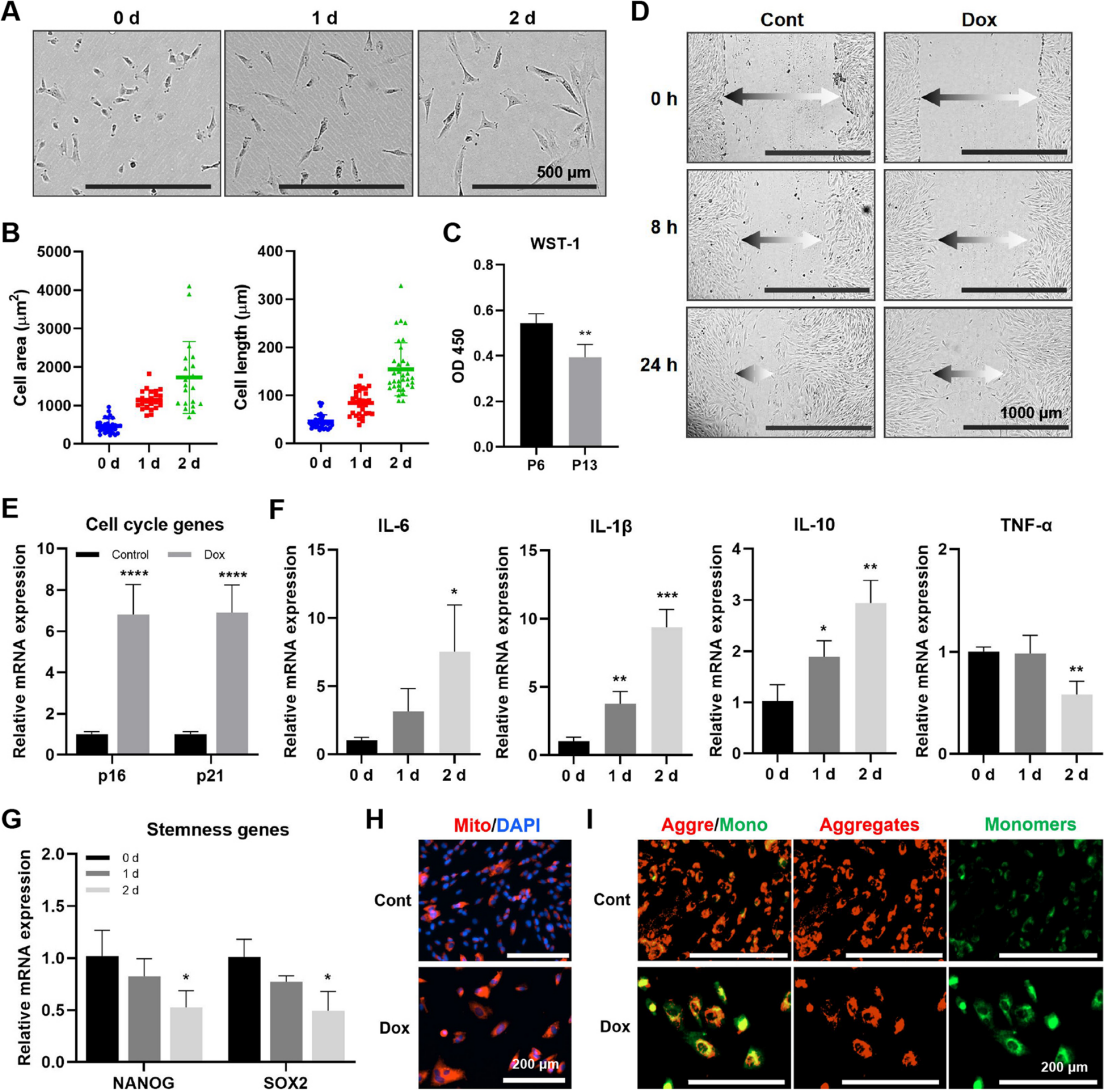
Changes of senescence-related indicators of iMSCs in doxorubicin-induced acute senescence. **A** Representative morphology of iMSCs in doxorubicin incubation. Scale bar: 500 μm. **B** Mean cell area and length increased with doxorubicin duration. **C** Proliferation of iMSCs with doxorubicin incubation. **D** Scratch assay to detect iMSC migration in doxorubicin incubation. Scale bar: 1000 μm, the arrow indicated the width of the scratch. The mRNA levels of cell cycle genes (**E**), inflammatory cytokines (**F**), and stemness genes (**G**) of iMSCs with doxorubicin incubation. **H** The mitochondrial density of iMSCs with doxorubicin incubation. Scale bar: 200 μm. **I** The MMP of iMSCs with doxorubicin incubation. Scale bar: 200 μm. Data were representative of three independent experiments. *n* = 3, * *p* < 0.05; ** *p* < 0.01; *** *p* < 0.001; **** *p* < 0.0001 by Student’s *t* test

Cell senescence is a cellular response triggered by various endogenous and exogenous factors, such as DNA damage, telomere dysfunction, and oxidative stress [35]. To induce acute senescence in iMSCs, we utilized doxorubicin, a widely used chemotherapeutic agent known for its ability to disrupt double-strand DNA breakage and cause extensive DNA damage [36]. In the presence of doxorubicin, early senescent iMSCs exhibited significant changes in cell morphology within a short period of 1 to 2 days, characterized by increased cell area and length (Fig. 3A,B). Furthermore, doxorubicin incubation resulted in decelerated cell migration (Fig. 3C), decreased cell proliferation (Fig. 3D), upregulated expression of senescence-related genes (Fig. 3E), altered expression of inflammatory cytokines (Fig. 3F), downregulated expression of stemness-related genes (Fig. 3G), increased mitochondrial density (Fig. 3H), and altered MMP (Fig. 3I).

**Fig. 4:**
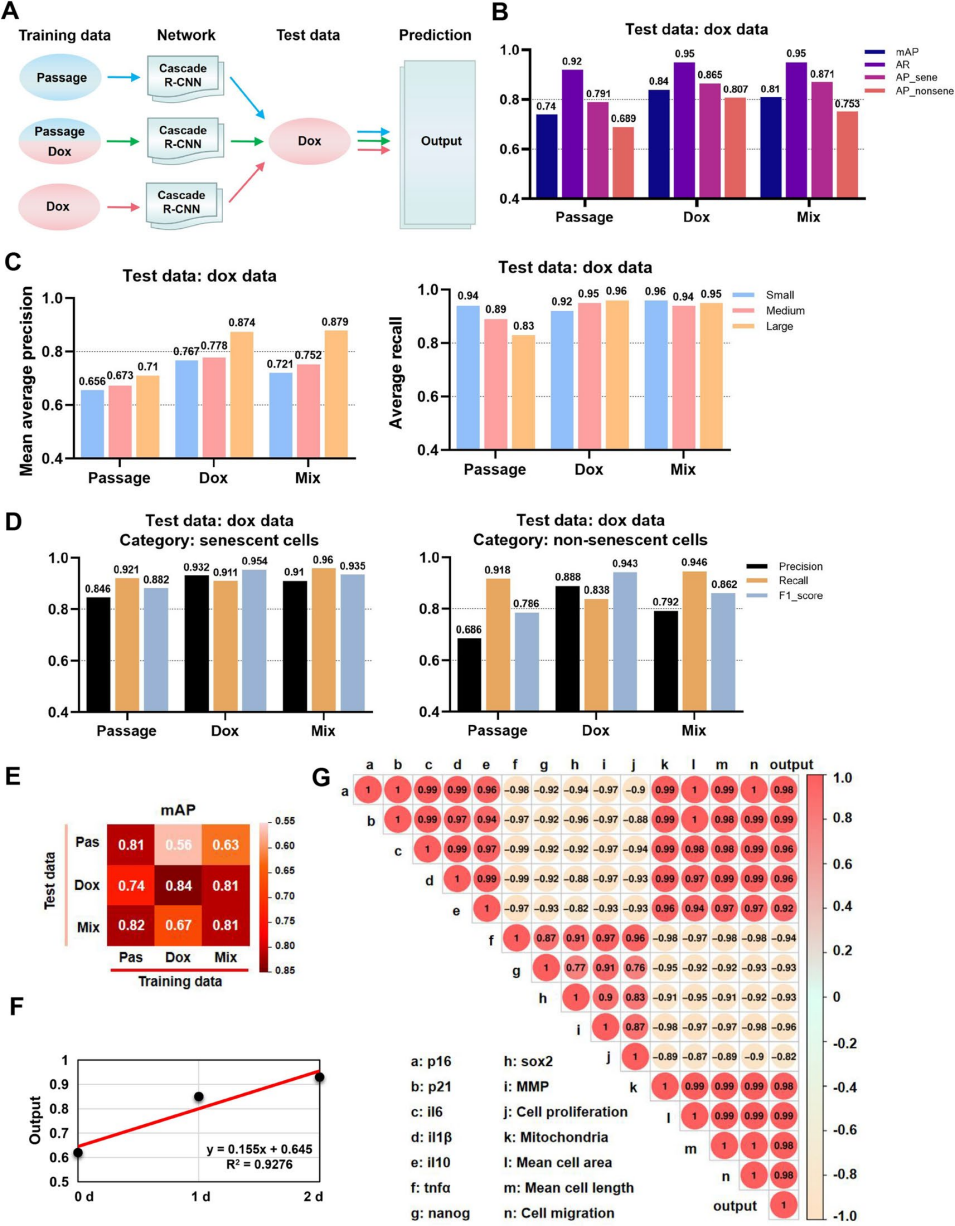
Cascade R-CNN system performance at doxorubicin-induced iMSC senescence. **A** Protocol for the development of the Cascade R-CNN in doxorubicin-induced iMSC senescence. **B** The AP, mAP, and AR showed the performance of the trained Cascade R-CNN trained in doxorubicin data. **C** The mAP and AR for small, medium, and large objects in doxorubicin data. **D** The precision, recall, and *F1* score for non-senescent and senescent cells detection in doxorubicin data. **E** A heatmap showed the mAP of Cascade R-CNN prediction in each test dataset. **F** Linear correlation between the senescence proportion output of Cascade R-CNN trained by doxorubicin data and the doxorubicin duration. **G** The Pearson correlation coefficient between the senescence proportion output of the Cascade R-CNN trained by doxorubicin data and senescence-related indicators. Data were representative of three independent experiments, *n* = 3

To validate the ability of the Cascade R-CNN network to identify senescent cells within the doxorubicin-induced senescent dataset, a new dataset was acquired and not utilized for network training. The input dataset comprised 640 × 640 pixels from brightfield images at × 4 magnification, with 219 images allocated for training and 67 images for testing. In the dataset, the training set consisted of 6110 senescent cells and 1324 non-senescent cells, while the test set contained 1584 senescent cells and 368 non-senescent cells. Doxorubicin-induced senescence was independent three times to enhance data generalization, and the doxorubicin-induced senescence was validated by SA-β-gal activity. Subsequently, three distinct Cascade R-CNN networks were evaluated using datasets of passage-induced senescence, doxorubicin-induced senescence, and mixed passage- and doxorubicin-induced senescence (Fig. 4A). Among the three different trained models, the Cascade R-CNN trained on both doxorubicin-induced senescence and mixed passage- and doxorubicin-induced senescence datasets exhibited superior performance in detecting doxorubicin-induced senescence images, with mAP values exceeding 0.81 and AR values surpassing 0.95 (Fig. 4B). Furthermore, we observed that these three distinct Cascade R-CNN networks demonstrated varying levels of efficacy in identifying small, medium, and large objects: for small objects, the average of mAP and AR were 0.714 and 0.94; for medium objects, the average of mAP and AR were 0.734 and 0.927; for large objects, the average of mAP and AR were 0.821 and 0.913, respectively (Fig. 4C). Compared to non-senescent cells, the three Cascade R-CNN networks exhibited exceptional performance in senescent cells, with averaged precision, recall, and *F*1 score of 0.896, 0.931, and 0.924 respectively (Fig. 4D). These finding provided strong support for the effectiveness of the Cascade R-CNN system in detecting doxorubicin-induced senescent iMSCs, highlighting its superior detection precision for larger object.

After this, we prepared three new datasets for testing, and each condition was independently repeated three times. The Cascade R-CNN trained using passage data exhibited exceptional performance across all three test datasets, with an average mAP of 0.79. Conversely, the Cascade R-CNN trained on the doxorubicin dataset exhibited superior performance exclusively in doxorubicin test images. The Cascade R-CNN, trained using a combination of passage and doxorubicin images, exhibited exceptional performance in both the passage and mix test datasets, with an average mAP of 0.75 (Fig. 4E and Fig. S2). During senescence, the spindle-shaped morphology of MSCs gradually transitions to an irregular shape. The morphological alterations of iMSCs exhibit distinct patterns during chronic and acute senescence. In chronic senescence, senescent and non-senescent MSCs do not represent absolute opposites; instead, a transitional state exists between them. Conversely, in drug-induced acute senescence, most non-senescent MSCs bypass the intermediate transitional state and promptly enter the late senescence state. Compared to doxorubicin, the images of the passage dataset contained more cell morphological features, which might have contributed to the poor performance of doxorubicin-trained Cascade R-CNN in the passage dataset.

The Pearson correlation coefficient revealed a highly linear correlation between senescence proportion output and the doxorubicin duration (Fig. 4F). The correlation between the senescence proportion output and other senescent-related parameters in doxorubicin data showed the strong positive correlations between senescence proportion output and cell cycle genes (*p16* and *p21*), inflammatory cytokines (*IL-6*, *IL-1β*, and *IL-10*), mitochondrial density, cell area, cell length, and migration, and strong negative correlations between senescence proportion output and stemness genes (*NANOG* and *SOX2*), *TNF-α*, MMP, and cell proliferation (Fig. 4G). These findings suggested that the morphometry-based Cascade R-CNN system could reliably identify drug-induced senescent cells.

### Cascade R-CNN system performance at senolytic-treated iMSC senescence

**Fig. 5:**
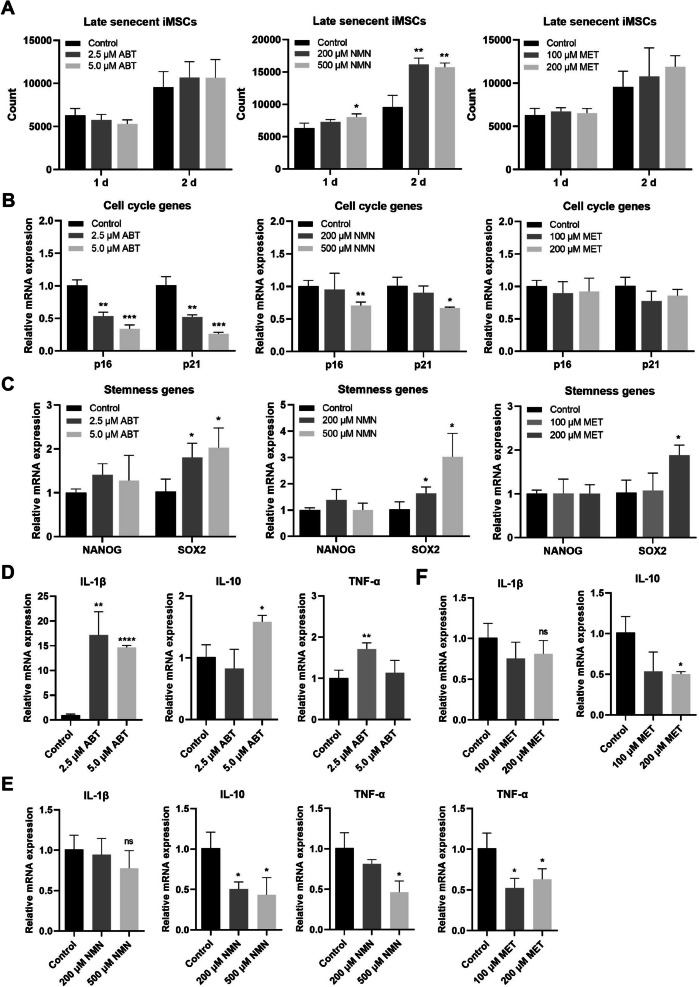
Effects of senolytics on iMSC proliferation and gene expression. **A** Changes of iMSC proliferation after senolytic incubation (ABT-263, NMN, and metformin). The expression of cell cycle genes (**B**), stemness genes (**C**), and inflammatory cytokines (**D–F**) of iMSCs with senolytic incubation (ABT-263, NMN, and metformin). Data were representative of three independent experiments, *n* = 3. * *p* < 0.05; ** *p* < 0.01; *** *p* < 0.001; **** *p* < 0.0001 by Student’s *t* test

**Fig. 6:**
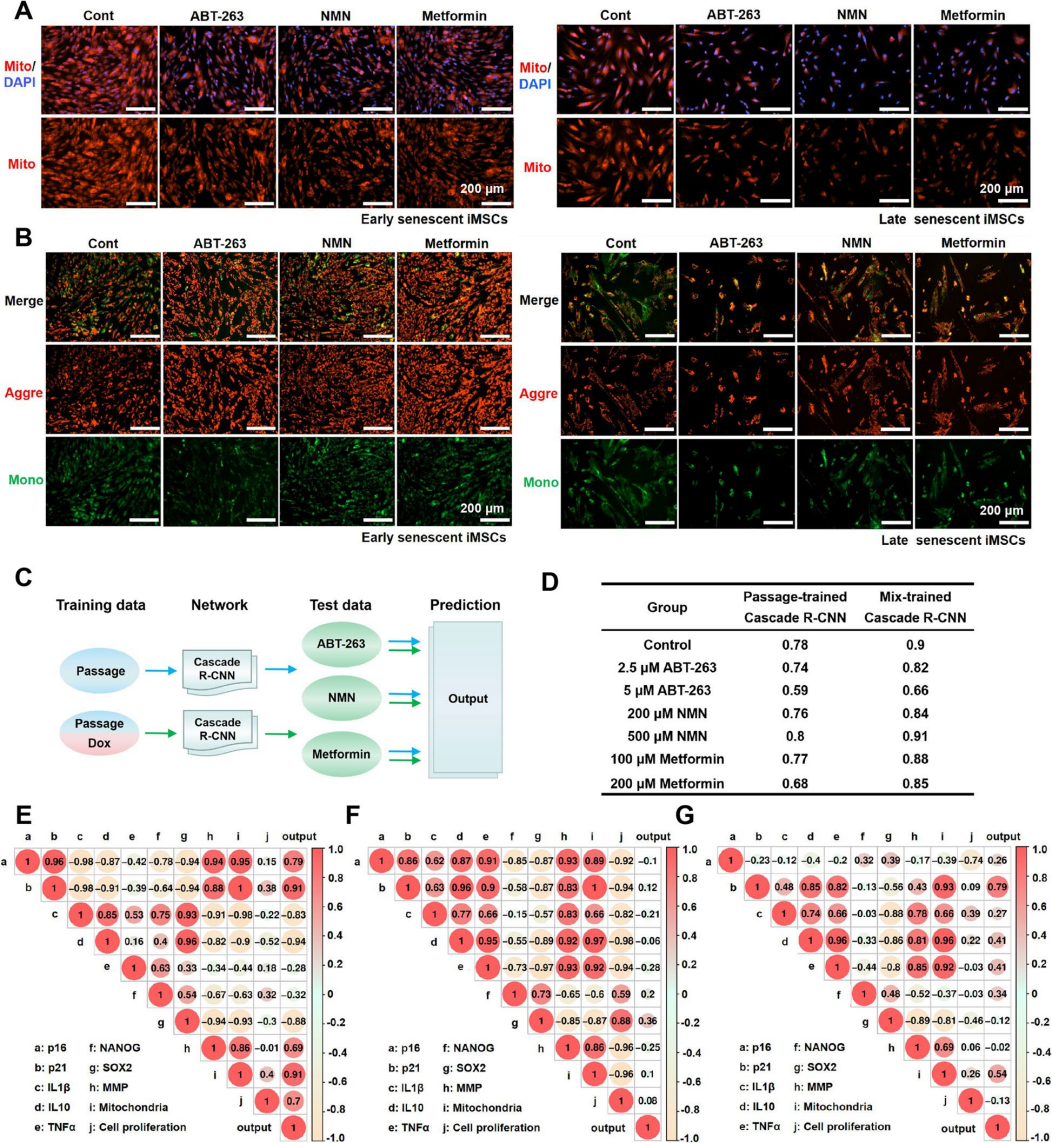
Cascade R-CNN system performance at senolytic-treated iMSC senescence. **A** The mitochondrial density of early and late senescent iMSCs with senolytic incubation. ABT-263, 2.5 μM; NMN, 200 μM; metformin, 100 μM. Scale bar: 200 μm. **B** The MMP of early and late senescent iMSCs with senolytic incubation. ABT-263, 2.5 μM; NMN, 200 μM; metformin, 100 μM. Scale bar: 200 μm. **C** Protocol for the Cascade R-CNN for detecting senolytic-treated late senescent iMSC data. **D** Output of the Cascade R-CNN to detect senolytic data. The Pearson correlation coefficient was calculated between the senescence proportional output of Cascade R-CNN and senescence-related indicators in the ABT-263 (**E**)/NMN (**F**)/metformin (**G**) incubation group. Data were representative of three independent experiments, *n* = 3

Senolytics have been proposed as potential therapeutic agents for age-related diseases due to their specific effects on senescent cells [37, 38]. In order to validate the performance of the Cascade R-CNN system, we investigated the impact of senolytics such as ABT-263, nicotinamide mononucleotide (NMN), and metformin on senescent iMSCs. By analyzing the effects of three drugs on iMSCs in late senescence, we found that only NMN promoted proliferation of senescent iMSCs, while ABT-263 and metformin did not have this effect (Fig. 5A). Both ABT-263 and NMN (but not metformin) reduced mRNA expression of *p16* and *p21* (Fig. 5B). All three drugs upregulated *SOX2* gene expression with little effect on *NANOG* gene expression (Fig. 5C). The mRNA expression of *IL-1β*, *IL-10*, and *TNF-α* was upregulated by ABT-263 (Fig. 5D), while NMN and metformin downregulated the mRNA expression of *IL-10* and *TNF-α* (Fig. 5E,F). All three drugs exhibited a reduction in mitochondrial density in late-senescent iMSCs (Fig. 6A), with ABT-263 demonstrating greater effectiveness on MMP (Fig. 6B).

The Cascade R-CNN system, trained on a dataset of passage-induced senescence or mixed passage- and doxorubicin-induced senescence, was used to analyze images following incubation with ABT-263, NMN, and metformin (Fig. 6C). Analysis by the Cascade R-CNN system trained on the passage-induced dataset revealed that the proportion of senescent cells decreased from 78 to 59%, 78 to 76%, and 78 to 68% for ABT-263, NMN, and metformin respectively. The Cascade R-CNN system, trained on a mixed dataset, analyzed images of ABT-263, NMN, and metformin and observed changes in the proportion of senescent cells from 90 to 66%, 90 to 84%, and 90 to 85%, respectively (Fig. 6D). Additionally, we employed ImageJ software analysis to quantify changes in cell length and area after senolytics treatment, revealing that ABT-263 had a greater effect on cell morphology compared to NMN and metformin treatments (Fig. S3). These results indicated that the three senolytics had different effects on iMSC morphology, among which ABT-263 showed the most promising outcomes.

The Pearson correlation coefficient indicated that in the ABT-263 incubation group, the senescence proportion output was a positive correlation with cell cycle genes (*p16* and *p21*), mitochondrial density, MMP, and cell proliferation, a negative correlation with inflammatory cytokines (*IL-1β* and *IL-10*) and *SOX2*, and weak correlation with *TNF-α* and *NANOG* (Fig. 6E). In the NMN, the senescence proportion output was a negative correlation with cell cycle genes (*p16* and *p21*), inflammatory cytokines (*IL-1β*, *IL-10*, and *TNF-α*), stemness genes (*NANOG* and *SOX2*), mitochondrial density, MMP, and cell proliferation (Fig. 6F). In the metformin, the senescence proportion output was a positive correlation with *p21*, inflammatory cytokines (*IL-10*, and *TNF-α*), and mitochondrial density, and weak correlation with *p16*, *IL-1β*, stemness genes (*NANOG* and *SOX2*), cell proliferation, and MMP (Fig. 6G). These results suggested that the three senolytics had different effects on indicators associated with cell senescence.

## Discussion

The presence of senescent cells should be avoided during the production of cell products due to their potential impact on cell quality and safety [39, 40]. Therefore, monitoring the state of cells is crucial for ensuring the production of high-quality and safe cell products. Cell morphology can serve as a distinctive marker for identifying different cell types and pathological states based on its morphodynamics, which include changes in protein structure and expression as well as chromatin structure. Researchers use morphology to identify the cell state but often produce uncertain detection results when faced with mass microscopic observation tasks. Therefore, the development of automated recognition and localization technology for cell images is crucial to facilitate the optimal utilization of morphological indicators. Oja et al. utilized the Cell Omics Morphology Explorer imaging system (version V4, Thermo Scientific) to quantify morphological alterations of MSCs during prolonged culture, including parameters such as width, length, perimeter, area, and shape. The study demonstrated that cell morphology serves as an indicator of cell quality and could be employed in developing novel non-invasive imaging-based techniques for screening and quantifying senescence in cell cultures [41]. Kusumoto et al. utilized a morphology-based CNN system to detect senescent cells, including human umbilical vein endothelial cells (HUVECs) and human diploid fibroblasts (HDFs), in phase-contrast microscope images. The senescence proportion output of pre-trained CNN was used to evaluate the quality of endothelial cells [42]. However, the Cell Omics Morphology Explorer imaging system has exhibited misclassification of overlapping cells as a single entity or fragments as intact cells [41]. Kusumoto et al. conducted automatic single-cell cropping on phase contrast microscope images beforehand and utilized the clipped single-cell image data for CNN training and detection, resulting in improved classification performance [42]. The accuracy of image segmentation often has an impact on classification performance. In multicellular images, the cellular features exhibit slight variations, boundaries are often indistinct, cells may overlap, and broken cells or impurities can cause interference, thereby increasing the complexity of image segmentation in traditional image detection. The deep learning-based object detection algorithms enable simultaneous prediction of the location and category of cells in an entire image, facilitating the detection of multicellular images in complex and microscopic scenes. Xu et al. achieved excellent performance in cervical cell smear images using the Faster R-CNN model, surpassing CenterNet and YOLOv5 algorithms in terms of time efficiency, recognition precision, and adaptability [43]. Moallem et al. used CNN and Faster R-CNN to achieve remarkable results in detecting patient-derived cancer cells in blood sample images, with Faster R-CNN proving more efficient and suitable for deployment [44]. Cascade R-CNN, which is based on Faster R-CNN, has been applied to various object detection tasks [25, 45]. However, it remains unclear whether Cascade R-CNN can effectively evaluate cell morphology with blurred boundaries in bright-field microscopy images. We have developed a method to evaluate the senescence-related state of MSCs based on cell morphology using Cascade R-CNN and assessed its performance in chronic and acute senescence. Our results demonstrated, for the first time, that this network can be reliably applied to detect MSC senescence in bright-field microscopy images. Compared to other commonly used object detection networks, Cascade R-CNN demonstrated superior performance. The senescence proportion outputs were then correlated with senescence-related parameters, such as cell proliferation, gene expression, and mitochondrial function, indicating that the network’s ability to assess the MSC senescence trend was comparable to the traditional biological method.

Development techniques for the identification of individual senescent cells can offer powerful monitoring capabilities for the application of reagents targeting senescent cell removal and the subsequent production of high-quality, highly stable therapeutic MSC products. Conventional methods relying on senescence biomarkers (such as telomere length, p16, mitochondrial damage) might cause irreversible cellular damage or modification, or exhibit delays during the detection process. The β-galactosidase-based assay could effectively identify individual senescent cells and serve as a reliable positive control for other methods. However, this method was found to be destructive and prone to false positives [46]. By integrating cell size measurement technology, the accuracy of identification could be significantly enhanced [41, 47]. The combination of cell size and spectral measurement [48, 49] offers the advantage of being non-destructive and label-free. However, the excitation light required for spectral measurement may cause photodamage to cells. Therefore, factors such as cell culture conditions and media must be taken into account when applying this technique. The detection system established in this study had obvious advantages over traditional biological and spectroscopic techniques when applied to single-cell analysis in microscopic images. Specifically, the system was characterized by its online detectability, rapidity, and reliability in evaluating senescent cell populations during two-dimensional planar culture without compromising subsequent cell production. The detection system enables researchers to perform analysis on × 4 bright-filed MSC images. The MSC images were clipped to 640 × 640-pixel and subsequently input into the system. The predicted category of each cell within the image could be obtained by running the code of the system.

For live cell imaging, we opted for the traditional six-well culture plate to capture images that more closely mimic the in vitro conditions of MSC. Only distortion-free areas containing cells were included in our imaging data collection. In this study, Cascade R-CNN demonstrated the ability to predict objects of varying sizes on feature maps at multiple scales, resulting in improved detection performance and reduced computational cost. However, the detection system exhibited a significant decrease in precision across cell culture generations, potentially due to differences in cell morphology and non-object background regions. The transition from non-senescence to senescence occurred gradually, and the morphology of cells in the intermediate state may confound detection. To address these issues, future research could incorporate more abundant training data and introduce adaptive threshold in transfer learning to extract domain-invariant features of MSCs across different passages. This would improve network precision and provide a more reliable choice for automating non-invasive evaluation of MSC quality.

In this study, we observed significant variations in the effects of three senolytics (ABT-263, NMN, and metformin) on iMSC senescence. Previous studies have indicated distinct mechanisms of action for three senolytics concerning cell senescence. ABT-26 inhibits B cell lymphoma 2 (BCL-2) and BCL-extra-large (BCL-X_L_), thereby inducing apoptosis of senescent cells through mitochondria-dependent pathways and mitigating aging at an individual level by reducing the presence of senescent cells and cytokines [50]. NMN, as a precursor of nicotinamide dinucleotide (NAD^+^), can safely and effectively increase NAD^+^ content, thereby alleviating cell senescence [51]. Metformin reduces AMP-activated protein kinase (AMPK)-independent SASP production by directly regulating nuclear factor-κB (NF-κB) binding and activation to achieve the anti-aging goal [52]. Senolytics may improve some senescence-related indicators without rapidly and effectively reversing cell morphology. Therefore, we posit that the morphology-based approach can serve as a rapid and cost-effective option for screening anti-aging drugs, albeit not a definitive choice. Nonetheless, this method still offers advantages in detecting senescent cells while saving labor and costs.

## Conclusions

In this study, we developed a Cascade R-CNN system for the detection of senescent MSCs, exhibiting promising performance in both replicative- and drug-induced senescence. This method, when combined with optical light microscopy capable of recording, presents a labor-saving and cost-effective option for screening culture conditions and anti-aging drugs for MSCs. Moreover, it offers an excellent technique for integrating non-invasive real-time morphological image analysis into cell production.

## Methods

### Cell cultures

The iMSCs utilized in this study were derived from induced pluripotent stem cells (iPSCs) (Nuwacell, Cat #RC1001, China), following the previously reported methodology [53]. To characterize the iMSCs, flow cytometry was employed to analyze the presence of typical MSC markers. The iMSCs were cultured in an incubator at 37 °C and 5% CO_2_ using MSC basal medium (Dakewe, China) supplemented with EliteGro™-Advanced serum-free supplement (Dakewe, China).

Doxorubicin (APExBIO, USA) was utilized to induce cell senescence in iMSCs. Specifically, iMSCs were cultured in MSC basal medium supplemented with serum-free supplement and 100 nM doxorubicin for 2 days to obtain senescent iMSCs exhibiting altered morphology. Control samples consisted of iMSCs cultured in MSC basal medium supplemented with a serum-free supplement and PBS for 2 days.

Senolytics (ABT-263, NMN, and metformin; Absin, China) were employed to modulate the senescent phenotype of iMSC. Specifically, iMSCs (passages 12–14) were treated with these three drugs for 2 days each.

### Cell proliferation

The cells were seeded onto a culture plate and cell proliferation was detected using the WST-1 cell proliferation and cytotoxicity assay kit (Beyotime Biotechnology, China). After adding 0.1 volume of water-soluble tetrazolium (WST) reagent, the culture medium was incubated at 37 ℃ for 2 h and absorbance was measured at a wavelength of 450 nm using a microplate reader (Bio-Tek, USA).

After staining the cells with diamidino-phenyl-indole (DAPI, 10 μg/mL, Life Technologies, USA), the culture wells were meticulously examined and captured under a microscope (Lionheart™ FX automated live cell imager with augmented microscopy™, × 4 objective, BioTek, USA), followed by precise segmentation and quantification of DAPI using advanced image analysis software (Gen5 Imager).

### Cell differentiation

#### Osteogenic differentiation

Human iMSCs were cultured until reaching 80–90% confluence, following which the MSC basal medium was substituted with osteogenic differentiation medium (Biological Industries, Israel). The differentiation process lasted for 21 days and necessitated a change of medium every 2 days. Visualization of the differentiation was achieved through Alizarin Red S staining (MSC osteo-staining kit, Vivacell, China) under a microscope.

#### Chondrogenic differentiation

The iMSCs were cultured unit reaching 80–90% confluency, following which the MSC basal medium was substituted with chondrogenic differentiation medium (Biological Industries, Israel). The differentiation process lasted for 21 days and necessitated a change of medium every 3 days. Visualization of the differentiation was achieved through Alcian blue 8GX staining (MSC chondro-staining kit, Vivacell, China) under a microscope.

### Cell migration

The serum-free medium culture was initiated when iMSCs reached over 90% confluence in the cultured well. Following serum starvation, a scratch was created in each well by scraping cells with a sterile plastic pipette tip. The cells were washed twice to remove cellular debris and then treated with 100 nM doxorubicin, while the control group received PBS. Images were captured using a microscope, and three independent scratch wound-healing assays were performed.

### Quantitative real-time PCR (qPCR)

Total RNA was extracted using a miRNA kit (Omega Scientific, USA). The cDNAs were synthesized with the ReverTra Ace qPCR RT master mix with gDNA Remover (Toyobo, Japan), and gene expression was quantified using the ChamQ SYBR qPCR master mix kit (Vazyme, China) on the LightCycler 480 Detection System (Roche, Switzerland). Each experiment was performed in triplicate. Data were analyzed using the 2^−△△Ct^ method with glyceraldehyde-3-phosphate dehydrogenase (*GAPDH*) as an internal control. The primer sequences used are listed in Table S1.

### Immunofluorescence

Prior to fixation in 4% paraformaldehyde at 37 °C for 15 min, the cells were stained with mito-tracker reagent (200 nM, Beyotime Biotechnology, China) for mitochondria and lyso-tracker reagent (50 nM, Beyotime Biotechnology, China) for lysosomes. The nucleus was stained with DAPI and visualized under fluorescence microscopy. Fluorescence intensity was quantified by calculating the integrated density using ImageJ software.

### JC-1 assay

The MMP was assessed using the JC-1 mitochondrial membrane potential assay kit (Beyotime Biotechnology, China). Before incubation with a medium containing JC-1 staining solution for 20 min at 37 °C, iMSCs were washed with PBS and subsequently analyzed via fluorescence microscopy. Quantification of fluorescence intensity was performed using ImageJ software.

### Sample preparation for the image analysis

Senescent cells were detected using an SA-β-gal staining kit (Beyotime Biotechnology, China) and stained with an SA-β-gal staining solution. Briefly, cells were washed thrice with PBS, fixed in 4% paraformaldehyde for 15 min at room temperature, and incubated overnight at 37 ℃. The activity of SA-β-gal was observed under a conventional light microscope.

Images were acquired using a Lionheart™ FX automated live cell imager equipped with augmented microscopy™ (× 4 objective, BioTek, USA). Three wells of the six-well plate were captured for each analysis, resulting in 360 images per run. The imaging was performed in a spiral mode starting from the center of each well to minimize optical distortions caused by bulges along the bottom edge. The length and area of each cell in the image were quantified using ImageJ software.

The training and test datasets were subjected to senescence induction through serial culture and doxorubicin incubation. A total of 7373 images were acquired from more than three independent experiments, with each image being saved as a 640 × 640-pixel RGB image in PNG format.

### Server and analysis environment

We used an Nvidia GTX Titan XP server with 4 CPUs: 6 Graphics Processing Clusters, 36 Texture Processing Clusters, 72 Streaming Multiprocessors, 4608 CUDA Cores, 576 Tensor Cores, 72 RT Cores, 1350 MHz Base Clock (MHz), 1770 MHz Boost Clock (MHz), 7000 MHz Memory Clock, 24 GB GDDR6 Total Video Memory. We wrote all the scripts on the Linux system using Python. LableMe, an open-source image labeling software, was used for data labeling [54].

### Training by deep learning methods

For training, we employed the Cascade R-CNN network [25], which incorporates a residual neural network (ResNet) as the baseline network and integrates feature pyramid network (FPN), region proposal network (RPN), and group normalization (GN) modules. ResNet enhances training depth by introducing residual units into deep neural networks. The inclusion of the FPN module facilitates improved feature extraction, while the RPN module significantly accelerates detection box generation for region proposals. Moreover, the GN module normalizes input images to further enhance detection accuracy.

Cascade R-CNN used cascade regression as a resampling mechanism to address the overfitting issue during training and the intersection over union (IoU) mismatch problem during inference, resulting in significant performance gains. The input images were fed into ResNet for feature map extraction, which was then used by the RPN module to generate region candidate boxes. Subsequently, the RoI (region of interest) coordinates were obtained. The corresponding features of the Rol region were forwarded to the pooling layer to acquire local feature maps with fixed height and width information. The local feature maps were input into distinct detection heads for classification and bounding box regression. During the training process, the IoU thresholds of the three detection heads were set as 0.5, 0.6, and 0.7, respectively. Subsequently, the local features generated by each detection head were forwarded to the pooling layer of the subsequent detection head (with increasing IoU values), ultimately resulting in outputting both detection boxes and classification categories. The cross-entropy error was utilized as the loss function, with a learning rate of 0.01 and an iteration count of 1000. Momentum optimization was applied at a value of 0.9, while L2 regularization was set to 0.0001. The criterion for stopping training was when the loss function stabilizes and reaches a minimal relative value. Each round of training, which corresponds to one epoch, involved the processing of all images.

The trained network was capable of producing the object category and its corresponding score (i.e., the confidence score of identifying non-senescent cells or abandoned cells). The performance of the network after training was evaluated using precision, recall, *F*1 score, precision-recall (PR) curve, AP, mAP, and AR.

For training using alternative deep learning methods, we utilized the same dataset of passages as employed in Cascade R-CNN training. The identification of iMSCs in images was conducted through SSD [28], CenterNet [29], FCOS [30], YOLOv3 [31], PicoDet [32], Deformable DETR [33], and Sparse R-CNN [34]. The networks (SSD [28], CenterNet [29], FCOS [30], YOLOv3 [31], PicoDet [32], Deformable DETR [33], Sparse R-CNN [34]) were trained with the optimal parameters reported in previous studies. These network performances were assessed based on AP and mAP metrics and subsequently compared to the performance achieved by Cascade R-CNN. The complete code used in this study can be found in the public GitHub repository, [https://github.com/PaddlePaddle/PaddleDetection/tree/release/2.6/configs].

The trained Cascade R-CNN networks were evaluated using newly collected datasets, including three independent experiments: passage-induced senescence, doxorubicin-induced senescence, and a combination of both. Each dataset was obtained from each of the three independent experiments. Network evaluation was conducted using precision, recall, *F*1 score, AP, mAP, and AR metrics.

### Evaluation of network performance

For network evaluation, precision, recall, *F*1 score, PR curve, AP, mAP, and AR were utilized with mAP50 as the test index. The IoU is employed to assess the accuracy of the detection box by evaluating the overlap between the detection box and the true box by calculating their intersection-to-union ratio. The IoU threshold of our model was 0.5 to judge the correctness of the detection results. The precision denotes the ratio of correctly classified positive samples to all samples classified as positive, thereby primarily reflecting the predictive accuracy of the results. The recall represents the proportion of correctly classified positive samples out of all true positive categories, primarily reflecting the rate of missed detections in the predicted results. The *F1* score considers the combined harmonic mean of precision and recall. The AP is calculated by integrating the area under the PR curve and serves to measure the performance of the detection algorithm across various categories. Ranging from 0 to 1, a higher AP value indicates better model performance in that category. The mAP, a widely adopted comprehensive evaluation metric in object detection tasks, is computed as the average of individual APs across all categories and serves as a performance measure for models across multiple categories. AR is the average of the recall of all test images within a specific interval, which is used to evaluate algorithm performance at different recall rates and serves as the standard for assessing missed detections. The mAP50 represents the index settlement of mAP at an IoU threshold of 0.5.

1 $$Precision=\frac{TP}{TP\,+\,FP}$$

2 $$Recall=\frac{TP}{TP\,+\,FN}$$

3 $$F1\ score=\frac{2\ Recall\,\times\, Precision}{Recall\,+\,Precision}$$

* TP: true positive, FP: false positive, FN: false negative.

### Correlation analysis

The correlation between the senescence proportion output of the Cascade R-CNN system and other senescence-related markers was assessed by calculating the Pearson correlation coefficient. Statistical planning and graphical analysis were performed using GraphPad Prism 8.4.3, while visualization of the correlation was achieved through heat maps and principal component analysis.

### Statistical analysis

The representative experimental data were also reported, and all data were presented as means ± standard deviation (S.D.). Each experiment was performed in triplicate using triplicate biological samples unless otherwise specified. The statistical significance of differences between the two experimental groups was analyzed using a *t*-test. *p* < 0.05 was considered statistically significant.

### Supplementary Information

**Additional file 1:** **Fig. S1.** Cascade R-CNN system performance at each passage data. **Fig. S2.** Cascade R-CNN system performance at passage data. **Fig. S3.** Cell area and length of senolytic-treated senescent MSCs. **Table S1**. Sequences of primers used for this study.

## Data Availability

The datasets supporting the conclusions of this article are available from the corresponding author upon reasonable request. The complete code of Cascade R-CNN can be found in the public GitHub repository, [https://github.com/PaddlePaddle/PaddleDetection/tree/release/2.6/configs/cascade_rcnn].

## Abbreviations

AMPK: AMP-activated protein kinase
AP: Average precision
AR: Average recall
BCL-2: B cell lymphoma 2
BCL-X_L_: BCL-extra-large
Cascade R-CNN: Cascade region-based convolution neural network
DAPI: Diamidino-phenyl-indole
Deformable DETR: Deformable detection transformer
Deformable R-FCN: Deformable region-based fully convolutional network
FCOS: Full convolutional one-stage object detection
FN: False negative
FP: False positive
FPN: Feature pyramid network
GAPDH: Glyceraldehyde-3-phosphate dehydrogenase
GN: Group normalization
HDF: Human diploid fibroblast
HUVEC: Human umbilical vein endothelial cell
IL: Interleukin
iMSC: Induced pluripotent stem cell-derived mesenchymal stem cell
IoU: Intersection over union
iPSC: Induced pluripotent stem cell
mAP: Mean average precision
MMP: Mitochondrial membrane potential
NAD: Nicotinamide dinucleotide
NF-κB: Nuclear factor-κB
NMN: Nicotinamide mononucleotide
P6: Passage 6
P13: Passage 13
PR: Precision-recall
qPCR: Quantitative real-time PCR
R-CNN: Region-based convolution neural network
ResNet: Residual neural network
RoI: Region of interest
RPN: Region proposal network
SA-β-gal: Senescence-associated β-galactosidase
SASP: Senescence-associated secretory phenotype
S.D.: Standard deviation
SSD: Single shot multibox detector
TNF-α: Tumor necrosis factor-alpha
TP: True positive
WST: Water-soluble tetrazolium
YOLOv3: You only look once v3
