# Supplementary material for: Morphology-based deep learning enables accurate detection of senescence in mesenchymal stem cell cultures
Source: BMC Biol. 2024 Jan 2;22:1. doi: 10.1186/s12915-023-01780-2 (PMC10762950; doi:10.1186/s12915-023-01780-2)
Supplement: Supplementary file 1 — Additional file 1: Fig. S1. Cascade R-CNN system performance at each passage data. Fig. S2. Cascade R-CNN system performance at passage data. Fig. S3. Cell area and length of senolytic-treated senescent MSCs. Table S1. Sequences of primers used for this study. [file 12915_2023_1780_MOESM1_ESM.doc]

**Supplemental information**

**
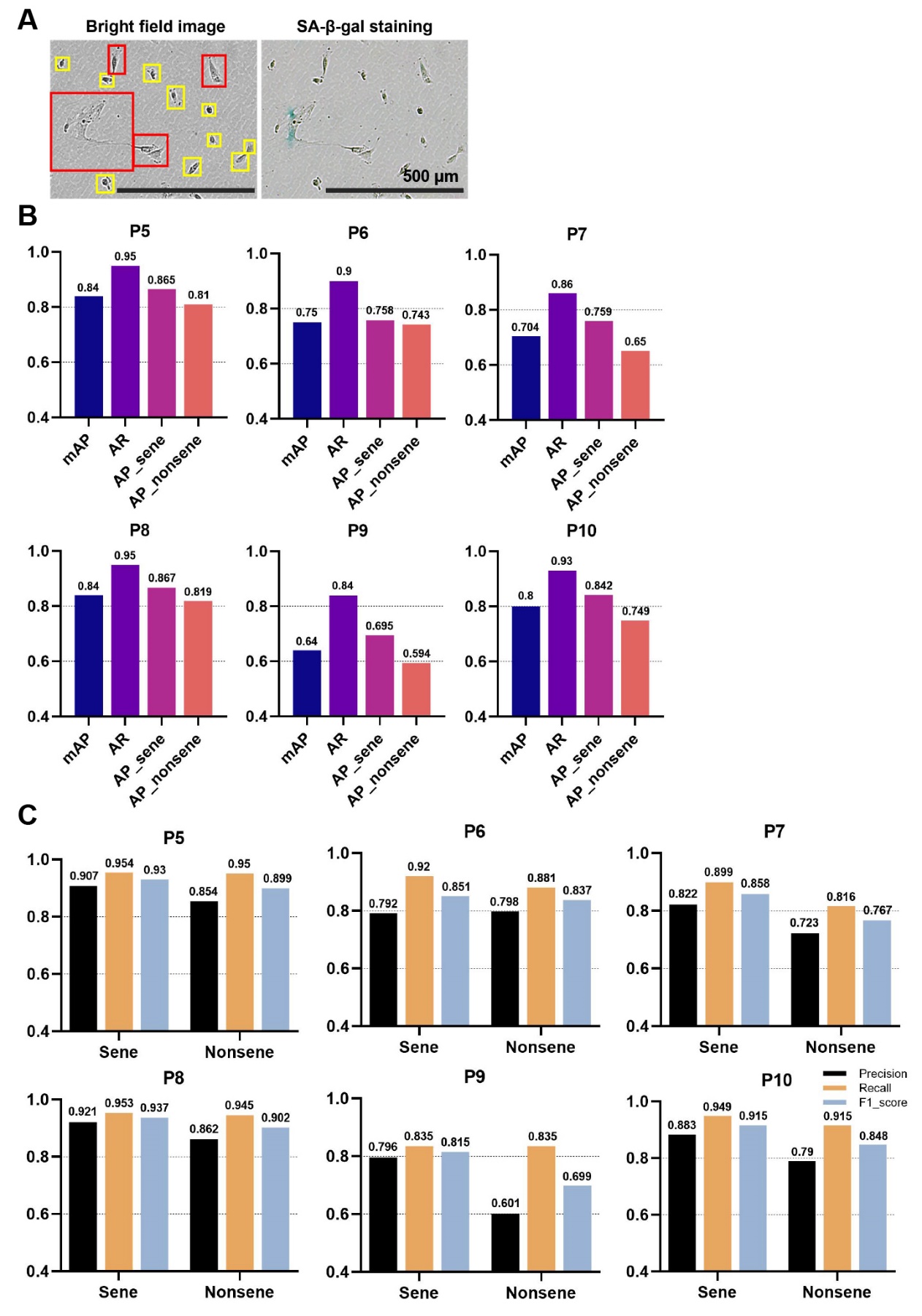
**

**Fig. S1** **Cascade R-CNN system performance at each passage data.**

A. Concept of images labeled according to SA-β-gal staining and cell morphology. The red boxes were labeled as senescent cells and the yellow boxes were labeled as non-senescent cells. Scale bar: 500 μm. B. The AP, mAP, and AR showed the performance of the Cascade R-CNN trained by passage data. C. The precision, recall, and *F1* score for non-senescent and senescent cell detection in each passage data. Data were representative of three independent experiments, *n* = 3.

**
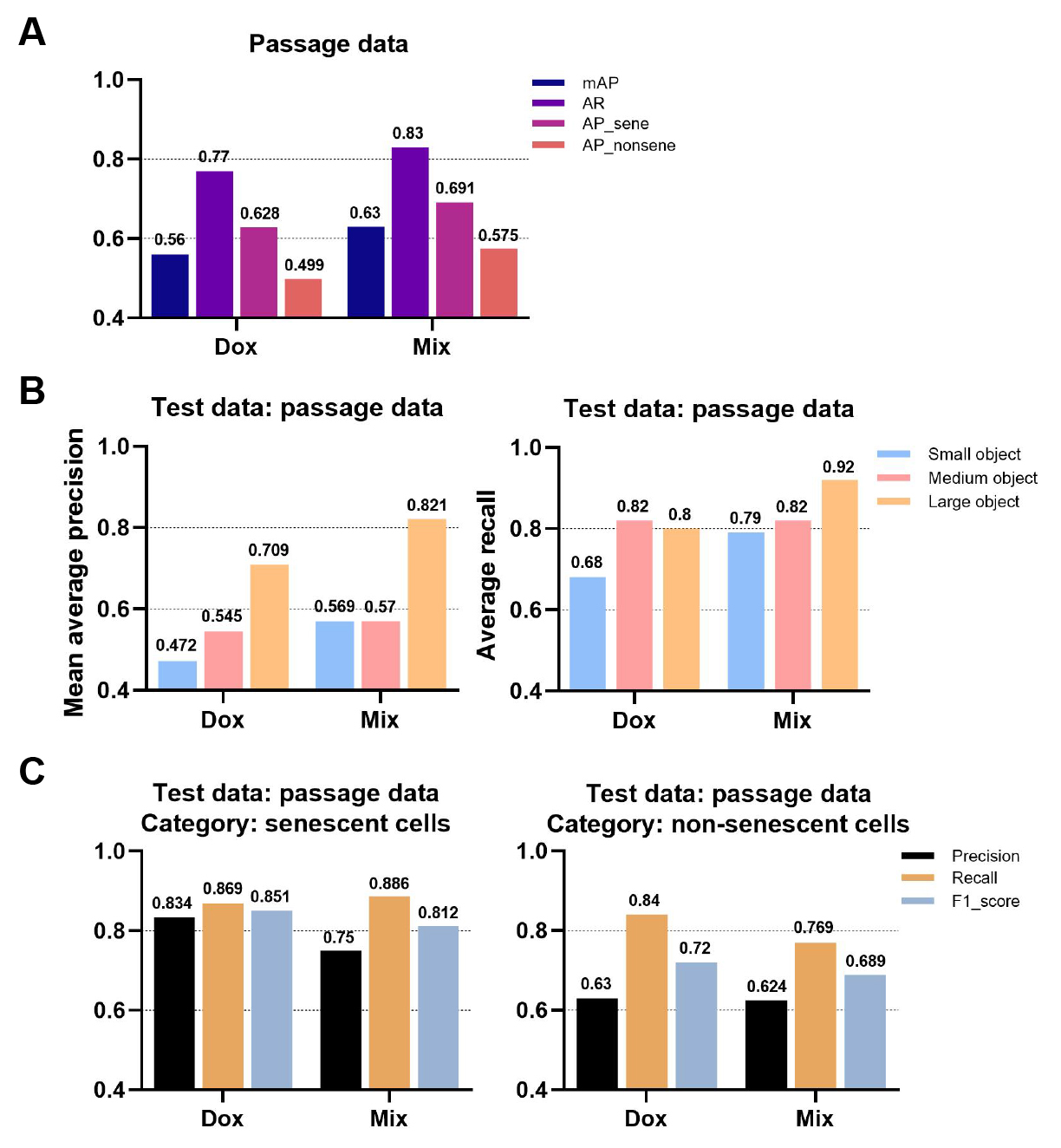
**

**Fig. S2 Cascade R-CNN system performance at passage data.**

A. The AP, mAP, and AR showed the performance of the Cascade R-CNN system. B. The mAP and AR for small, medium, and large objects in passage data. C. The precision, recall, and *F1* score for non-senescent and senescent cells detection in passage data. Data were representative of three independent experiments, *n* = 3.

**
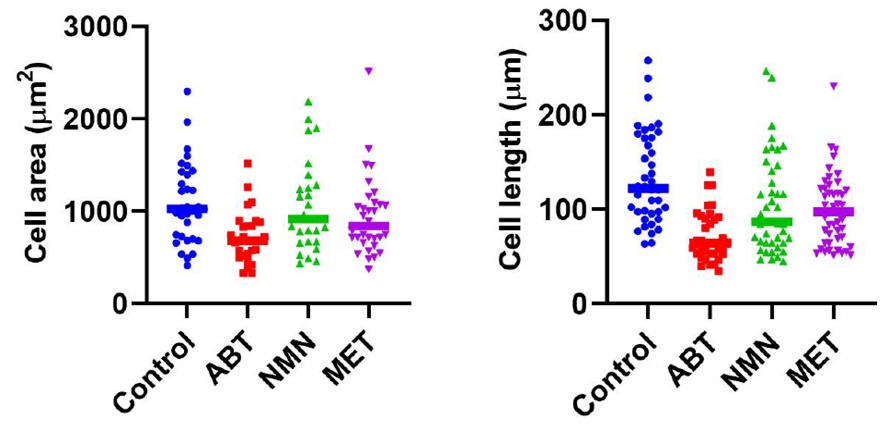
**

**Fig. S3 Cell area and length of senolytic-treated senescent MSCs.**

**Table S1**

Sequences of primers used for this study.

| Primer | Sequence (5′ - 3′) |
| --- | --- |
| p16-F | AGGGGACAGCAGAGGAAG |
| p16-R | GCGTTTGGAGTGGTAGAAATCTG |
| p21-F | GAGGCCGGGATGAGTTGGGAGGAG |
| p21-R | CAGCCGGCGTTTGGAGTGGTAGAA |
| NANOG-F | GATGCCTCACACGGAGACTG |
| NANOG-R | GCAGAAGTGGGTTGTTTGCC |
| SOX2-F | GACAGTTACGCGCACATGAA |
| SOX2-R | TAGGTCTGCGAGCTGGTCAT |
| IL-6-F | GAACTCCTTCTCCACAAGCG |
| IL-6-R | TTTTCTGCCAGTGCCTCTTT |
| IL-1β-F | CCACAGACCTTCCAGGAGAATG |
| IL-1β-R | GTGCAGTTCAGTGATCGTACAGG |
| IL-10-F | AAGCCTGACCACGCTTTCTA |
| IL-10-R | ATGAAGTGGTTGGGGAATGA |
| TNF-α-F | TGGCCAATGGCGTGGAGCTG |
| TNF-α-R | GTAGGAGACGGCGATGCGGC |
| GAPDH-F | GAAGGTGAAGGTCGGAGT |
| GAPDH-R | GAAGATGGTGATGGGATTTC |
